# The History and Capabilities of Herbal Simples

**Published:** 1890-02-22

**Authors:** W. T. Fernie


### The History and Capabilities of Herbal Simples.

By W. T. Fernie, M.D.

*(Continued from page 293.)*

### V.—THE COMMON ARUM, ASPARAGUS, AND BALM.

### Common Arum.

In the days of our childhood we have all played with the "lords and ladies" of English fields and moist hedgerows, which plant is so called from the colour, either purple or white, of its erect pointed spike standing within the curling hood of an arrow-shaped emerald leaf. Botanically, this herb is the Arum Maculatum of the natural order Araceæ. It further bears the popular names of Cuckoo-pint, Wake Robin, Parson-in-the-Pulpit, Calf's Foot, Ramp, Starchwort, and Gethsemane. Its tuberous root has been analysed, and found to contain a particular volatile acrid principle, which exercises distinct medicinal effects, but which is altogether dissipated when the roots are baked or boiled. The plant also contains gum, sugar, starch, and fat. Its fresh juice yields malate of lime, and when tasted causes a burning, stinging irritation of the mouth and throat; also, if swallowed, it will produce cracked lips, with a raw, red state of the tongue. The leaves, when applied externally to a delicate skin, will blister it. A medicinal tincture is made of the root with spirit of wine, and of this from five to ten drops may be given for a dose, with a tablespoonful of cold water, three times a day to an adult. It is curative of a feverish, sore mouth, and of the affection called clergyman's sore throat. For a chronic sore throat, with vocal hoarseness and swollen mucous membrane, the tincture will generally be found of essential service; likewise for a tendency to irresistible sleepiness and heaviness after a principal meal; as also for chronic rheumatism. An ointment made by stewing the fresh sliced root with lard proves efficient in curing ringworm. The name Arum is derived from the Hebrew "jaron"—a dart, in allusion to the shape of the leaves, like spear heads, or, as some think, from "aur"—fire—because of the acridity of the juice. The adjective, "maculatum," refers to the dark spots and patches which are seen on the smooth, glossy leaves of the plant. These leaves have frequently proved fatal to children, who have mistaken them for sorrel. The brilliant, coral-like berries which are found set closely about the erect spike of the arum in the autumn are known to country lads as adders' meat, a name corrupted from the Anglo-Saxon word "attor"—poison—as originally applied to these berries. Mr. Grant Allen justifies their highly poisonous character by suggesting that cock robins, deluded by the scarlet prettiness of the berries, devour them, and die speedily. Then the bodies of the infatuated birds soon decay, and form a mouldering heap of manure, from which the young cuckoo-pint presently derives a ready store of rich nutriment. Moreover, experience does not serve to deter other robins in the future from repeating this act of folly, seeing that the same bird can never eat the poisonous berries twice, and that its survivors hold no coroner's inquest over its body to inquire into the cause of its death. Though, indeed, we were assured in our nursery days, on veracious authority, that "All the birds in the air fell to sighing and sobbing when they heard of the death of poor Cock Robin!" It is a curious fact that pheasants can eat the red berries of the arum with complete impunity. In Germany a superstition still holds good that when a young man goes to a dance, if he will put a piece of arum in his shoe, he will secure for himself the most eligible partners in the room. Recently a patented drug named "Tonga" has obtained considerable public favour as specific against obstinate neuralgia of the head and face. On the supposition that this nostrum is got from the stem of a species of Raphidophora, which belongs alike to the order of arums, some fresh juice expressed from the common arum maculatum has been tried in a severe case of neuralgia, which was previously relieved only by Tonga, and it was found that this juice, in teaspoonful doses, gave similar relief. The name "Starchwort" was given to the Arum in Queen Elizabeth's time, because its roots were then used for supplying pure white starch to stiffen the frills and ruffs which were worn by gallants and ladies. The "British Domestic Herbal" of Sydenham's day relates a case of alarming dropsy, with great constitutional exhaustion, treated by him most successfully with a medicine of arum and angelica, which cured in three weeks.

### Asparagus.

The Asparagus, of which when cultivated the tender shoots are eaten, grows wild on the coasts of Essex and Suffolk and Cornwall. It is then a more prickly plant than when nurtured and manured in our gardens. Its name comes from the Persian "spurgas," a shoot, and it belongs to the lily order of plants. The Greeks and Romans valued it as a vegetable, and they boiled it so quickly that to be done "sooner than asparagus"—"velocius quam asparagi coquuntur"—became a proverb with them, to which our modern phrase, "done in a jiffey," closely corresponds. The juice of the shoots, whether wild or cultivated, contains wax, albumen, phosphate, and acetate of potash, mannite, a green resin, and a particular principle named asparagin. This asparagin stimulates the kidneys and gives a peculiar strong smell to the urine soon after taking the shoots, whilst the green resin exercises gentle sedative properties calculated to calm nervous excitability, palpitation, and the like. A medicinal tincture is made from the young plant with spirit of wine, and of this eight or ten drops may be taken for a dose, with a tablespoonful of cold water, three times a-day, for irritable urinary passages, also for rheumatic gout. The crystalline principle, asparagin, may be given in powder for passive dropsy of the heart. A syrup of asparagus is employed medicinally in France, and at Aix-les-Bains it forms part of the cure for rheumatic patients to eat asparagus. Gerard calls the plant sperage, "which is easily concocted, when eaten, and doth gently loose the belly." The feathery green growth of the mature asparagus plant, generally supposed to be branched leaves, really consists only of ramified flower stalks, which do duty for leaves. They have been substituted by the plant when growing wild on arid, sandy soils, where no juicy moisture could be got for the maintenance of leaves. These shrubby stalks of the asparagus bear red, coral-like berries, which when ripe contain grape-sugar and spargancin. They are attractive to small birds, who swallow them whole, and afterwards void the seeds, to germinate when disseminated in this way. Thus it would seem there is more valid reason than the mere vulgar corruption of the proper name, Asparagus, for calling this plant familarly sparrowgrass or grass. Botanically, it may be regarded as a lily which has seen better days, and which is sowing its wild oats late in life.

### Balm.

The herb Balm—Melissa—which is quite commonly cultivated in our cottage gardens, yields from its leaves and tops an essential oil, containing a chemical—*stearopten*. The name Balm is an abbreviation of balsam, which signifies "the chief of sweet-smelling oils," and the botanical suffix, melissa, bears reference to the large quantity of honey (mel)

contained in the flowers of this herb. The wild, or bastard balm, is called mellitis, and grows in woods in the south of England. Each belongs to the Labiate order of plants. Bawme, say the Arabians, makes the heart merry and joyful. "The juice thereof," says Gerard, "glueth together greene wounds." And the leaves, say both Pliny and Dioscorides, being applied, "do close up woundes without any perill of inflammation." It is now known as a scientific fact that the balsamic oils of aromatic plants make most excellent surgical dressings. They give off ozone, and thus exercise anti-putrescent effects. Moreover, as chemical "hydrocarbons," they contain so little oxygen that in wounds dressed with the fixed balsamic herbal oils the atomic germs of disease are starved off. Furthermore, the resinous parts of these balsamic oils, as they dry upon the sore or wound, seal it up, and effectually exclude all noxious air. So the essential oils of balm, peppermint, lavender, and the like, with pine oil, resin of turpentine, and the balsam of benzoin (Friar's balsam) should serve admirably as domestic applications by being bound on lint or fine rag over cuts and superficial sores. To ambulance volunteers the lamentation of Jeremiah should fail now to give discouragement, "Is there then no balm in Gilead; is there no physician there?" By reason of the volatile oil which the herb contains, balm tea, a justly popular simple, if taken hot, promotes perspiration on the access of catarrh or influenza; also, if similarly used, it will aid effectively in bringing on the delayed monthly flow with women. But a cold infusion of the plant, made with cold water, acts better as a remedy for hysterical headache and as a general nervine restorative. Formerly a spirit of balm, combined with lemon-peel, nutmeg, and angelica root enjoyed a great reputation as a restorative cordial under the name of Carmelite Water. Paracelsus thought so highly of balm that he believed it gave promise to completely renovate man—"primum eus melissae. "The London Dispensatory," 1696, said: "The essence of balm, given in canary wine every morning, will renew youth, strengthen the brain, relieve languishing nature, and prevent baldness." The "balm in Gilead" referred to above was formerly of great esteem in the East as a medicine and as a fragrant unguent. It was the true balsam of Judaea, which at one time grew nowhere else in the whole world than at Jericho. But when the Turks took the Holy Land they transplanted this balsam to Grand Cairo, and guarded its shrubs most jealously by janissaries during the time the balsam was flowing.

